# Iron imbalance in neurodegeneration

**DOI:** 10.1038/s41380-023-02399-z

**Published:** 2024-01-12

**Authors:** Sonia Levi, Maddalena Ripamonti, Andrea Stefano Moro, Anna Cozzi

**Affiliations:** 1 Vita-Salute San Raffaele University, Milano, Italy; 2 IRCCS San Raffaele Scientific Institute, Milano, Italy; 3 Department of Psychology, Sigmund Freud University, Milan, Italy

**Keywords:** Neuroscience, Physiology

## Abstract

Iron is an essential element for the development and functionality of the brain, and anomalies in its distribution and concentration in brain tissue have been found to be associated with the most frequent neurodegenerative diseases. When magnetic resonance techniques allowed iron quantification in vivo, it was confirmed that the alteration of brain iron homeostasis is a common feature of many neurodegenerative diseases. However, whether iron is the main actor in the neurodegenerative process, or its alteration is a consequence of the degenerative process is still an open question. Because the different iron-related pathogenic mechanisms are specific for distinctive diseases, identifying the molecular mechanisms common to the various pathologies could represent a way to clarify this complex topic. Indeed, both iron overload and iron deficiency have profound consequences on cellular functioning, and both contribute to neuronal death processes in different manners, such as promoting oxidative damage, a loss of membrane integrity, a loss of proteostasis, and mitochondrial dysfunction. In this review, with the attempt to elucidate the consequences of iron dyshomeostasis for brain health, we summarize the main pathological molecular mechanisms that couple iron and neuronal death.

## Introduction

Iron is essential for brain functions such as neuronal development, myelination, the synthesis and catabolism of neurotransmitters, electron transport and respiration [1]. The efficiency of Fe^2+^ ions as electron donors and Fe^3+^ ions as electron acceptors is fundamental for many biochemical reactions and makes iron indispensable for life. On the other hand, the same features that make iron useful make it toxic and dangerous. Indeed, iron is a strong promoter of oxygen radical species that can drive the oxidation of proteins, lipid peroxidation and nucleic acid modifications [2]. All these molecular alterations ultimately compromise vital cellular functions and could lead to cell death. An increase in reactive oxygen species (ROS) that overpowers the antioxidant capacity of the organism results in a condition known as oxidative stress, which is worsened by iron accumulation and can lead to faster tissue degeneration [3]. This mechanism has been observed in different pathologies characterized by primary or secondary iron overload. For these reasons, iron levels must be tightly regulated through adequate homeostasis pathways that allow cells to utilize iron by avoiding its harmful effects [4]. The basic mechanisms that regulate systemic iron have been elucidated, and these involve iron-dependent expression of liver hepcidin (Hep) and its interaction with ferroportin (Fpn) (excellent review on this topic in [5, 6]), while mechanisms that regulate brain iron are poorly known. In vivo magnetic resonance imaging (MRI) [7] and postmortem studies [8] revealed that total iron concentration increases with age in specific brain areas, but the reason why this increase is limited to some brain regions is still unclear. This physiological iron deposition during aging possibly contributes to senescence [9], while even higher iron accumulation occurs in the substantia nigra in Parkinson’s disease (PD) [10] and in anatomical regions affected by beta amyloid plaques and tau burden in Alzheimer’s disease (AD) [11], pointing to iron deregulation as a key player in the pathogenesis of common neurodegenerative diseases. The identification of rare monogenic disorders, named Neurodegeneration with Brain Iron Accumulation (NBIA) and characterized by severe iron accumulation in basal ganglia and extrapyramidal movement dysfunction (list in Table 1), has further provided evidence of how alterations in iron homeostasis are related to neurodegeneration [12, 13]. In addition, although iron deficiency is essentially associated with neurodevelopmental and neuropsychological disorders [14], the recent identification of new diseases caused by mutations in the *IREB2* gene, encoding for a protein involved in control of iron homeostasis and leading to brain iron deficiency and severe neurodegeneration, suggests a link between these last two phenotypes [15–17]. Thus, both iron overload and iron deficiency may trigger pathways leading to neuronal death, validating iron imbalance as a main cause of neurodegeneration. In this review, we first provide a brief description of brain iron metabolism. Then, we report the current knowledge on the molecular mechanisms related to iron dysregulation and neurodegenerative processes, describing some examples of the main pathological pathways triggering neurodegenerative diseases.

**Table 1:** List of NBIAs, main characteristics and relative references.

Gene	Disease	OMIM	Heritance	Function	Brain Iron	Clinical Features	Reference
*CP*	Acerulo-plasminaemia	#604290	AR	Iron oxidation	Basal ganglia	Movement disorders, dementia, retinal degeneration, dysarthria, ataxia	Miyajima H. 1987 [192]
*PANK2*	Pantothenate kinase-associated neurodegeneration (PKAN)	#234200	AR	Panthotenate phosphorylation; Coenzyme A synthesis	Globus Pallidus, “eye of the tiger sign”	Dystonia, spasticity, cognitive decline, pigmentary retinopathy	Zhou B. 2001 [142]
*FTL1*	Neuro-ferritinopathy	#606159	AD	Cellular iron storage	Basal ganglia, cerebellum, motor cortex, mild cerebral and cerebellar atrophy	Extrapyramidal movement disorders, parkinsonisms	Curtis A.R. J. 2001 [91]
*PLA2G6*	PLA2G6-associated neurodegeneration (PLAN)	#256600 #610217	AR	Hydrolysis of ester bonds at the sn-2 position of phospho-lipids; Membrane remodeling	Globus pallidus in <50% of cases	Infantile neuroaxonal dystrophy, hypotonia, gait disturbance, cerebellar atrophy. Dystonia, spasticity and parkinsonisms in adulthood	Morgan N.V. 2006 [193]
*SCP2*	Leukoencephalopathy with dystonia and motor neuropathy (LKDMN)	#613724	AR	Thiolase activity; Breakdown of branched chain fatty acids	Thalamus	Dystonia, spasmodic torticollis, spinocerebellar ataxia, balance and gait impairment	Ferdinandusse S. 2006 [194]
*ATP13A2*	Kufor-Rakeb disease (KRS)	#606693	AR	Lysosomal cation pump; autophagosome formation	Often no iron overload.	Parkinsonism, pyramidal signs, altered eye movements, dementia	Ramirez A. 2006 [195]
*DCAF17*	Woodhouse-Sakati syndrome (WSS)	#241080	AR	Protein associates with cullin 4/damaged DNA binding protein1 ubiquitin ligase complex	Sometimes iron overload in Globus pallidus and Substantia nigra	Extrapyramidal symptoms, dystonia, cognitive impairment, hypogonadism, alopecia, diabetes mellitus	Alazami A.M. 2008 [196]
*FA2H*	Fatty acid hydroxylase-associated neurodegeneration (SPG5)	#612319	AR	Hydroxylation of fatty acids; Ceramide synthesis; Myelin formation	Globus Pallidus, Substantia nigra	Profound ataxia, dystonia, dysarthria, spastic quadriplegia, axial hypotonia, optic atrophy	Kruer M.C. 2010 [197]
*C19orf12*	Mitochondrial membrane protein-associated neurodegeneration (MPAN)	#614298	AR/AD	Unknown; Lipid metabolism? Membrane remodeling?	Globus Pallidus, Substantia Nigra; abundant Lewy bodies	Global developmental delay, dystonia, parkinsonism, psychiatric symptoms, spastic paraparesis	Hartig M.B 2011 [198, 199]
*WDR45*	β-propeller-associated neurodegeneration (BPAN)	#300894	X-linked (de novo mutations)	Protein-protein interaction; Early autophagosome formation	Globus pallidus, substantia nigra	Global developmental delay, neurological deterioration, dystonia, parkinsonism cognitive decline, seizures,	Haack T.B. 2012 [200]
*COASY*	COASY protein-associated neurodegeneratin (CoPAN)	#615643	AR	4’-PP adenyltran-sferase and dephospho-CoA kinase; Coenzyme A synthesis	Globus Pallidus	Oro-mandibular dystonia, dysarthria, obsessive-compulsive behavior	Dusi S,2014 [201]
*GTPB2*	Jaberi-Helai Syndrome (JABELS)	#617988	AR	Unknown; mRNA/ribosome stability?	Globus pallidus, substantia nigra	Cerebellar atrophy. Mental retardation, ataxia, dystonia	Jaberi E. 2016 [202]
*REPS1*	NBIA7	#617916	AR	Endocytosis, vesicle transport	Globus pallidus, peduncles	Trunk hypotonia, progressive cerebellar ataxia, pyramidal syndrome. Cerebellar and cerebral atrophy	Drecourt A. 2018 [150]
*CRAT*	NBIA8	#617917	AR	Carnitine acetyltrasnferase, -oxidation	*G*lobus pallidus, substantia nigra	Cerebellar atrophy, posterior leukodystrophy	Drecourt A. 2018 [150]
*AP4M1*	Spastic paraparesis 50 (SPG50)	#612936	AR	Vesicle formation	Globus pallidus reported in a single family	Early-onset developmental delay, deterioration of motor function, tetraparesis, intellectual disability	Roubertie A. 2018 [203]

*AD* autosomal dominant, *AR* autosomal recessive.

## Iron in the brain

**Fig. 1:**
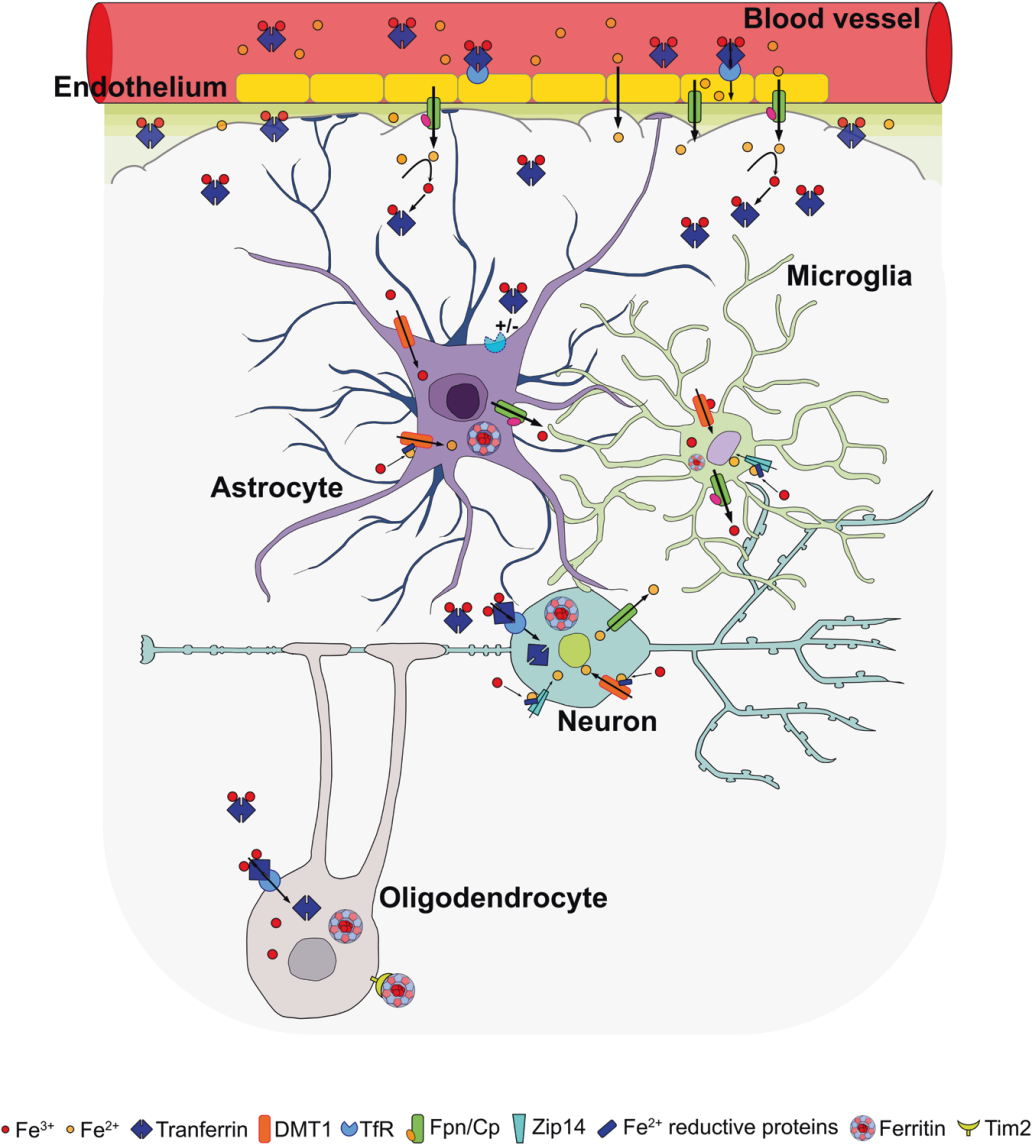
Cartoon depicting an example of iron transfer among different resident CNS cells and the different transporters involved. Iron enters BBB endothelial cells as Tf-TfR1 or via NTBI binding-mediated endocytosis. The ferric ion is thus released at the basolateral side by Fpn in the CNS interstitial fluid and associates with Tf, synthesized in the choroid plexus. NTBI is associated with ascorbate, citrate or ATP (released by astrocytes). Astrocytes internalize iron via DMT1, store it in ferritin, and distribute it to cells in the CNS via Ceruloplasmin-coupled Ferroportin (Cp/Fpn). Oligodendrocytes acquire metal through the ferritin receptor Tim-2 or DMT1. Neurons can acquire iron through the Tf-TfR1 pathway and DMT1.

**Fig. 2:**
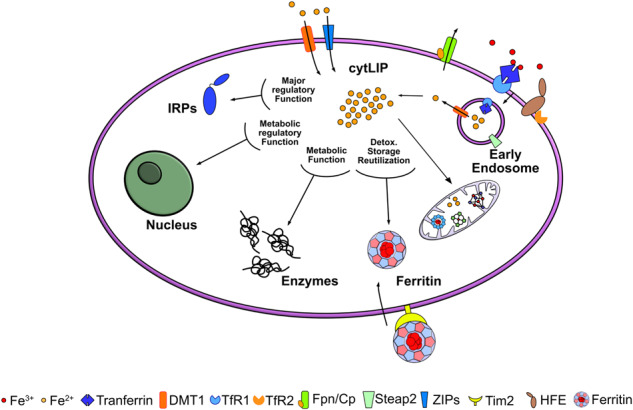
Main metabolic cellular pathways involved in iron homeostasis, usage, and transport. Iron incorporated into the cell, via Tf/TfR1 endocytosis or through DMT1/ZIPs, reaches the cytosol and mitochondria for support the ISC and heme biosyntheses. TfR2 form a complex with hemochromatosis protein, HFE, and serves as a component of the iron sensing machinery to regulate iron homeostasis. Fpn is the only iron-protein exporter involved in release of metal from the cell. The cytosolic labile iron pool (cytLIP), the redox-active iron available for the synthesis of iron enzymes, is in direct contact with only two classes of cytosolic proteins. They are highly represented and can bind iron: ferritins bind Fe-oxygen complexes, while IRPs link Fe-S (ISC) complexes. Ferritins store excess iron, and IRPs act as iron sensors.

The regional distribution of iron in a healthy adult brain is heterogeneous; the highest iron concentrations are detected in the basal ganglia (putamen, globus pallidus and caudate nucleus), while lower concentrations are detected in cortical gray matter, white matter, the midbrain and the cerebellum, and even lower iron concentrations are detected in the pons, locus coeruleus and medulla. The regional heterogeneity of brain iron was confirmed in vivo by MRI [7]. The main site that controls iron levels is the blood–brain barrier (BBB), structure that regulates iron transport from the blood stream to brain tissue. The endothelial cells of the BBB divide two distinct environments at their opposite surfaces, the basal and apical ends. The apical surface, which faces the blood stream, expresses the transferrin (Tf, a glycoprotein that binds and transports two iron atoms) receptor (TfR1). The absorption of transferrin-bound iron occurs through Tf/TfR1-mediated endocytosis by clathrin-coated vesicles (Figs. 1, 2). The different expression of TfR1 in distinctive regions of the brain represents the main cause of the uneven distribution of this metal. Indeed, the basal ganglia, substantia nigra and hippocampus show the highest expression of TfR1 compared to the cortex and brainstem [18]. Another TfR isoform exists, TfR2, but it has different functions [19, 20]; it has lower affinity (approximately 30-fold less) for iron-loaded transferrin and is involved in the regulation of systemic iron homeostasis by its interaction with HFE [19, 21]. Non-transferrin- bound iron (NTBI) can cross the BBB associated to various ligands, such as citrate, ATP, and albumin, located at the apical portion and probably internalized by vesicular endocytosis [22]. Alternatively, when NTBI is located near the apical surface of endothelial cells, it is reduced to ferrous iron by ferroreductases, including Steap 2 [23], and permeates the BBB thanks to DMT1 [24, 25] or other dimetal transporters, such as ZIP14, ZIP8, and L-type and T-type calcium channels [26]. The transport of NTBI across the BBB has long been controversial because there was no evidence of iron accumulation in patients affected by systemic iron overload such as subjects suffering from hemochromatosis and thalassemia, two diseases characterized by high serum levels of NTBI. More recently, some MRI studies on populations affected by thalassemia or haemochromatosis have highlighted the accumulation of iron in the brain of these subjects [27, 28]. Iron bound to ferritin, the iron-storage protein, can also permeate the BBB thanks to ferritin receptors such as Scara5 and Tim-2 [29, 30]. Iron entering the cell through DMT1 can be transferred to poly(rC)-binding protein 2 (PCBP2) [31], which acts as a chaperone and releases iron to cellular enzymes that need it [32, 33]. Once internalized, iron must reach the basal surface to be excreted in the CNS interstitial fluid and distributed throughout the brain. Cytosolic iron is exported into the interstitium *via* Fpn, an iron-exporter protein that appears to be expressed on both portions of the plasma membrane of BBB endothelial cells, suggesting that a portion of the cytosolic iron re-enters the systemic circulation [23, 34–37]. Ferrous iron, before being released into the interstitium by Fpn and binding to Tf, is oxidized to ferric ions by the action of ferroxidases, such as hephestin, which is produced by oligodendrocytes [38, 39], or by ceruloplasmin (Cp), which is produced by astrocytes and binds to the membrane thanks to a glycosyl phosphatidyl inositol anchor [40]. Oxidized iron enters the interstitial fluid of the CNS, where it binds Tf, which is synthesized by choroid plexus [41] and redistributes iron to cells exposed to the cerebrospinal fluid and interstitial fluid. Oligodendrocytes also synthetize Tf, but in vitro experiments on a human oligodendrocyte cell line showed the cytosolic localization of the protein and did not confirm the oligodendrocytes Tf secretion [42]. Another important site of iron entrance is the choroid plexus, where endothelial cells are permissive to the passage of different molecules with a filtering action carried out mainly by tight junctions on the apical layer of epithelial cells [41].

## Iron in CNS cells

Most CNS cells express a complete set of proteins involved in iron handling, such as TfR1 and DMT1 for iron import, H- and L- ferritin for metal storage, mitoferrin1 for mitochondrial metal replenishment, and Fpn as an iron exporter. The expression of these proteins is regulated at the post transcription level by the action of IRP1 and IRP2, which sense the level of intracellular iron and conveniently orchestrate the translation of iron responsive element (IRE)-containing mRNA for iron proteins (IRPs/IRE machinery) to maintain the optimal intracellular iron level (for a complete description of the mechanism, see [4, 5]). However, the expression of each protein varies according to the cell and to the amount of the metal present. For example, the level of cytosolic ferritin expression in brain cells varies according to the specific functional iron demands of different cell types. Neurons contain a fraction of it, while microglia contain the largest portion. Mitochondrial ferritin [43] is expressed only in highly ROS-sensitive neurons [44], as expected for a protein that has a fundamental role in protecting against oxidative stress [45–47]. Under physiological conditions, iron is mainly delivered to the mitochondria or utilized by cytosolic iron enzymes, and its excess is sequestered in ferritin to avoid ROS formation (better detailed in Fig. 2).

There is a continuous give and take of iron in the CNS among different resident cells, such as astrocytes, neurons, oligodendrocytes, and microglia however, the mechanisms by which this exchange occurs are not yet completely clarified. As demonstrated for systemic iron absorption [6], several studies have reported the importance of hepcidin/ferroportin (Hep/Fpn) interactions [48] in regulating these exchanges between brain tissue cells [49]. A fundamental role is played by astrocytes: they provide structural and metabolic support to neurons. In fact, they contact the BBB through membrane protrusions and establish direct synaptic-like connections with neurons. A recent work [50] defined the primary role of astrocytes in guiding iron transfer from blood to brain tissue. Through in vivo and in vitro experiments, the authors demonstrated that astrocytes respond to intracellular iron level variations by secreting hepcidin. Astrocyte-derived Hep, binding Fpn expressed by brain microvascular endothelial cells, regulates iron transport throughout the BBB [50]. Thus, astrocytes also play a key role in determining the amount of iron in brain tissue, resulting in an important model for the study of iron-dependent neurodegenerative diseases.

## Iron in aging and neuroinflammation

Aging processes lead to an increase in the amount of iron in brain tissue. This physiological process could compromise the iron homeostatic system [51], leading to an excess of iron that is not efficiently chelated by iron proteins. The increase in total iron concentration with aging could be caused by several factors, including increased permeability of the BBB [52, 53], the redistribution of iron within the brain and changes in iron homeostasis.

Other age-dependent changes relate to iron distribution among various molecules (ferritin, neuromelanin, transferrin, and others) in different cell types. In microglia and astrocytes of the cortex, cerebellum, hippocampus, basal ganglia and amygdala, ferritin concentrations generally increase with age. Oligodendrocytes contain the highest amount of iron, stored mainly as ferritin and transferrin, but their concentration remains constant with aging. In the aged brain, there is a subpopulation of ferritin-positive microglial cells [54], and most of these cells have an aberrant dystrophic morphology; iron is phagocytosed by ferritin-positive microglial cell subpopulations and likely becomes a source of toxic species that leads to cell degeneration. Thus, ferritin-positive, dystrophic microglia might contribute to the pathogenesis of neurodegenerative disorders due to impaired microglial function and can lead to region-specific increases in brain iron.

Detailed human studies have been performed in the substantia nigra and locus coeruleus to elucidate the effects of aging on iron, neuromelanin and ferritin accumulation [55, 56]. In healthy individuals, the total iron amount in the locus coeruleus remains stable throughout life and is lower than that in the substantia nigra, in which there is a linear increase in total iron concentration with age [56]. In the substantia nigra, the concentration of ferritin increases with age; thus, iron could contribute more to neurodegeneration in the substantia nigra than in the locus coeruleus. Additionally, the concentration of neuromelanin-iron complexes, which are the dominant form of iron in catecholaminergic neurons, increases with age in the substantia nigra and locus coeruleus. Again, the amounts of iron in the substantia nigra and globus pallidus are higher than those in other areas of the brain and may contribute to triggering the neurodegenerative process [57].

In addition, there is an increased proinflammatory state in the brains of older adults that results in a self-maintaining cycle of neuroinflammation and neurodegeneration [58]. Glial cell number increases in the normal aging brain, and there is an increase in the immunoreactivity markers of astrocytes and microglia [59]. Reactive macroglia secrete inflammatory mediators that reshape iron homeostasis, interfering with the activity of IRP1 and leading to iron accumulation [60]. Additionally, inflammatory stimuli via the upregulation of the iron homeostasis regulator Hep may stimulate an increase in iron and improve the detrimental cycle [61]. In a pro-inflammatory state, microglia uptakes NTBI and expands the ferritin storage pool, limiting extracellular iron. In an anti-inflammatory state, IL-4 increases the expression of TfR to promote the uptake of transferrin iron, resulting in ferritin degradation and iron release to support the activity of oligodendrocytes and neuronal regeneration [62]. However, this model oversimplifies the situation. In fact, microglial secretion of inflammatory cytokines like TNF-α and IL-1β increases neuronal iron uptake [62, 63], potentially leading to iron accumulation in neurons and, subsequently, cell death. Thus, iron and inflammation are interlocked in a bidirectional relationship (recent review on the topic [64, 65]) that was revealed to be present in many neurodegenerative diseases, e.g., PD [65], AD [66], HD [67], FRDA [68], and multiple sclerosis (MS) [69]. For example, in MS it has also been observed that iron is highly prevalent in the lesions [70]. This later work underlined that iron deposition in MS seems caused by regional distribution rather than an altered global brain iron load, suggesting brain iron redistribution as the origin of iron accumulation, at least in diseases associated with inflammation.

## Iron and cell death

Excess iron is strictly linked to cell death. The destructive influence of iron is due to its ability to catalyze the so-called Haber-Weiss reaction (O_2_^−^ + H_2_O_2_ → ·HO + HO^−^ +O_2_) within the cellular environment. This is a two-phase reaction: the first phase leads to the reduction of the ferric ion to the ferrous ion (Fe^3+^ + O_2_^−^ → Fe^2+^ + O_2_), and the second phase is called the Fenton reaction, which drives the formation of a highly reactive species represented by ·OH (Fe^2+^ + H_2_O_2_ → Fe^3+^ + OH^−^ + ·OH) that can oxidize cellular macromolecules, ultimately compromising vital cell functions and inducing cell death. For example, catecholamines, such as dopamine, can be oxidized to highly reactive or toxic quinones, either through the reduction of ferric iron or enzymatically [71]. Lipids are also easily subjected to oxidative modification by ROS with a particularly devastating process in lipid-rich brain tissue.

**Fig. 3:**
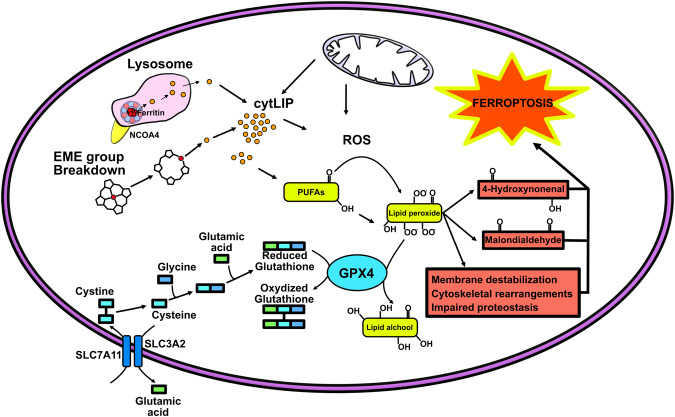
Graphic representation of the cellular mechanisms involved in the increase in ferroptotic events. Ferroptosis leads to membrane destabilization, mitochondrial dysfunction, cytoskeletal rearrangements, and protein impairment. It is triggered by an imbalance between lipid hydroperoxide detoxification and iron-dependent ROS accumulation. The peroxidation of Polyunsaturated fatty acids (PUFAs) is limited by glutathione peroxidase 4 (GPX4), which utilizing glutathione (GSH), converts the lipid hydroperoxide in lipid alcohol. When equilibrium is lost, the oxidized lipid species (4-Hydroxynonenal and Malondialdehyde) accumulate in membranes, destabilizing them and leading to cell death. SLC7A11, solute carrier family 7 member 11 and SLC3A2, solute carrier family 3 member 2 allow the internalization of cystine need for GSH synthesis. A key ferroptotic player is glutathione depletion and/or the inactivation of glutathione-dependent antioxidant enzyme GPX4. Source of iron are heme and cytosolic ferritin degradation. Under conditions of iron restriction, NCOA4 binds to the H-subunit of ferritin, carrying it to lysosomes (ferritinophagy), where the protein is degraded and iron is released; during iron excess, NCOA4 is degraded by the ubiquitin–proteasome system, making cytosolic ferritin free to sequester iron.

In 2012, Dixon et al. described a form of iron-dependent cell death that was named ferroptosis [72]. Ferroptosis is not a form of apoptosis, necrosis or autophagy, as it differs from them morphologically, genetically and biochemically [73]. Ferroptosis is defined as an iron-dependent regulated form of cell death characterized by the accumulation of lipid hydroperoxides (reviewed in [74]). The effects of ferroptosis include membrane destabilization, mitochondrial dysfunction, cytoskeletal rearrangements, and the impairment of protein degradation, all of which are detrimental to the cell (Fig. 3). A key player in the ferroptosis pathway is nuclear erythroid 2-related factor 2 (NRF2), a transcription factor that controls the expression of many antioxidant genes and components of ferroptosis [75]. More precisely, when NRF2 moves into the cell nucleus, it amplifies the transcription of a specific set of genes associated with detoxification and antioxidant reactions. These genes include heme oxygenase-1 (HO-1), NAD(P)H quinoline oxidoreductase, glutathione S-transferase superoxide dismutase-2 (SOD2), sulfiredoxin-1, H-ferritin, and various other antioxidant proteins [76]. Consequently, this helps in averting the accumulation of lipid hydroperoxides caused by ROS increment, preventing ferroptosis. Indeed, ferroptosis also requires glutathione (GSH) depletion and/or the inactivation of glutathione-dependent antioxidant enzyme glutathione peroxidase 4 (GPX4) [77, 78], a physiological controller of lipid hydroperoxide formation. Indeed, the depletion of *gpx4* in mice causes iron dysregulation, lipid peroxidation, hippocampal neurodegeneration and behavioral dysfunctions, suggesting that ferroptosis may be a key mechanism in AD diseases [79, 80]. Today, several studies have demonstrated that ferroptosis is closely related not only to the pathogenesis of AD but also to the majority of neurodegenerative diseases, such as Parkinson’s disease [81], HD [82], MS [83] and amyotrophic lateral sclerosis (ALS) [84]. In particular, in ALS where iron accumulation is visible in the corticospinal motor pathway before the onset of the disease and the detection of high ferritin levels in the serum is a negative predictor of the disease’s progression [85], iron alterations might trigger susceptibility to ferroptosis. A further work indicate that SEC24B, a regulator of COPII-mediated protein trafficking, is upregulated in this and in other neurodegenerative [86] disease. Curiously, this factor, identified as a novel regulator of ferroptosis, is particularly expressed in microglia [86]. These brain cells, containing high level of iron, have a major susceptibility to ferroptosis and exacerbate neuronal death.

## Iron-related impairment of protein degradative pathways and neurodegeneration

A further control step to avoid ferroptosis is the management of the amount of iron in the cell by ferritin. Ferritin can allocate approximately 4000 iron atoms/molecule inside its cavity; thus, it is the main source of iron for enzymatic requirements inside the cell. Intracellular iron recycling is physiologically maintained by ferritin degradation. This process is called ferritinophagy and involves iron-dependent nuclear receptor coactivator 4 (NCOA4) [87]. Under conditions of iron restriction, NCOA4 selectively binds to the H-subunit of ferritin and carries it to lysosomes to be degraded. After ferritin degradation, iron is resolubilized by the acidic pH of lysosomes and is released as Fe^2+^ into the cytosol through DMT1 or via the Ca^2+^/Fe^2+^-permeable channel TRPML1 [88]. Iron here is then reutilized to maintain cellular enzymatic activities. During iron overload, NCOA4 is degraded by the ubiquitin–proteasome system, leaving cytosolic ferritin free to store iron [89]. If iron exceeds the ferritin buffer capacity, free iron may induce ferroptosis [90]. Thus, ferritinophagy is a central process that controls intracellular iron levels and their detrimental effects.

A typical example is neuroferritinopathy (NF), a very rare autosomal dominant movement disorder belonging to the NBIA group (Table 1). NF is caused by mutations in *FTL1*, which encodes the L-ferritin subunit [91]. This subunit forms complexes with the H-ferritin subunit to form the heteropolymer ferritin [92]. The incorporation of the mutated subunit in ferritin heteropolymers results in a cytosolic increase in free redox-active iron due to the reduced ability of mutated ferritin to keep iron safely stored in its cavity [93–97]. NF patients show pathological iron deposition in different brain regions, especially in the globus pallidus [91, 98–100]. Analyses at a microscopic level showed iron overload in the nuclei and cytoplasm of oligodendrocytes, microglia and neurons; here, iron was frequently found to be bound in inclusion bodies containing wild-type and mutated subunits of ferritin [91, 99, 100]. These studies suggested that abnormal ferritin overexpression, aggregation and consequent proteostasis could be the primary cause of neurodegeneration, while the impairment of iron metabolism might occur as a secondary event [101, 102]. However, other important findings were obtained studying cellular models; these works provided evidence that the alteration of ferritin function drives cytosolic redox active iron to trigger a cascade of events leading to ferritin aggregation and the impairment of both proteasomal and lysosomal systems [93, 103]. Ultrastructural analysis of brains from NF transgenic mice confirmed the presence of iron–ferritin body complexes accompanied by signs of oxidative damage and revealed the impairment of the lysosomal compartment with the formation of lipofuscin. Lipofuscin, typical aging marker, is a pigment granule containing lipid residues of the lysosomal digestion and metal [94]. This evidence can explain the etiopathogenesis of human neuroferritinopathy [95]; moreover, new additional findings were obtained studying NF fibroblasts and induced pluripotent (iPS)-derived NPCs and neurons [104, 105]. The analysis of these models indicated that non-ferritin-bound iron causes the reduction of NCOA4, impairing ferritinophagy with consequent ferritin/iron aggregation, cell senescence and ferroptotic cell death. These results provide strong evidence supporting the primary role of iron in neuronal aging and degeneration [104]. In agreement, recently, the treatment of four NF patients with the BBB permeable iron-chelator deferiprone (DFP) resulted in a positive clinical outcome [106]. In one case, the authors were able to revert symptoms after a few months of treatment, showing that the earlier the treatment was initiated, the better the results on disease progression were. These results are promising, but further investigations are needed on a larger cohort of patients [106].

Iron excess is also pivotal in the pathogenesis of AD. In AD, there is an impairment in the metabolization of the amyloid beta precursor protein (amyloid precursor protein, APP) that triggers the formation of a neurotoxic molecule, β-amyloid, which slowly accumulates in the brain [107]. Several experimental studies have indicated that there is an interaction between iron metabolism and β-amyloid (Aβ) protein metabolism. First, APP contains an IRE, meaning that it can be post-transcriptionally regulated by the IRP/IRE machinery [108]. Thus, iron content determines the amount of APP [109], and iron also controls β-amyloid production by regulating the activity of furin, a member of the subtilisin-like convertase family [110]. Small amounts of iron increase the activity of furin, while high levels of cellular iron decrease the activity of this enzyme. Furin in turn, if active, induces α-secretase to stimulate the non-amyloidogenic pathway; in fact, high concentrations of iron inhibit furin, resulting in the production of β-amyloid. Studies in the brains of AD patients and Tg2576 mice have shown that the amounts of mRNA encoding furin are much lower than those in healthy controls [111]. In addition, it was suggested that APP binds Fpn, stabilizing it and allowing iron efflux from the cell [112, 113]. Furthermore, alterations in iron regulatory proteins such as transferrin, IRPs and ferritin have been observed. In patients carrying the APOε4 allele, the increase in ferritin detected in CSF was strongly associated with cognitive decline, indicating that iron imbalance can be one of the risk factors for AD [114]. Iron overload and oxidative stress in the brains of people with AD have been associated with the aggregation of beta-amyloid (Aβ)-induced senile plaque deposition [109, 115–117] and hyperphosphorylated tau proteins that form neurofibrillary tangles in the brain [11]. Iron-dependent phosphorylation and consequent tau protein aggregation occur not only in AD but also in all tauopathies, including PD, HD, PSP, frontotemporal dementia and others, that share iron accumulation as a common feature [118]. Moreover, iron excess also promotes the aggregation of α-synuclein protein, one of the main components of Lewy bodies in PD [119, 120].

## Iron-related mitochondrial dysfunction and neurodegeneration

**Fig. 4:**
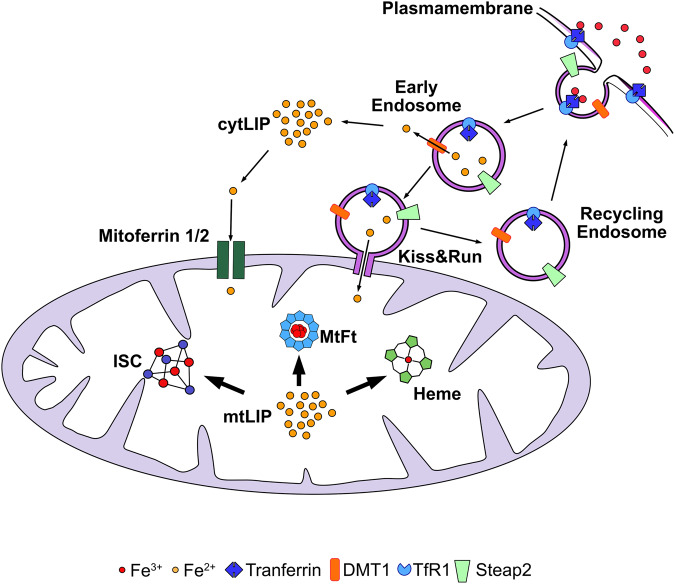
Cartoon depicting an example of iron uptake and utilization in mitochondria. Clathrin-coated endosomes containing TfR1-bound iron are endocytosed. The endosome lumen is acidified by a proton pump; the acidification decreases Tf-iron binding affinity, and as consequence, iron is released into the endosome lumen. Here, ferric ions are reduced by Steap2 and released through DMT1 into the cell cytosol. TfR1 is recycled back to the plasma membrane by recycling endosomes. Cytosolic free iron enters mitochondria through the mitoferrin channels. A second mechanism, called Kiss&Run, has been described to deliver iron to mitochondria, which consists of transient fusion between endosomes and mitochondrial membranes. Inside the mitochondrion, the labile iron pool (mitLIP), the redox-active form of iron, is used for sustaining heme and ISC biosynthesis or stored in mitochondrial ferritin (mtFt).

The relationship between mitochondrial dysfunction and neurodegeneration is often associated with Ca^2+^ dyshomeostasis [121, 122], but it must be considered that iron homeostasis is also fundamental for organelle functionality. Indeed, the mitochondrion plays a key role in cellular iron metabolism; it is the major iron-consuming organelle due to its need to sustain the biosynthesis of heme and iron-sulfur cluster (ISC) prosthetic groups, which are essential compounds for life [123, 124] (Fig. 4). The import of iron into mitochondria has been widely studied in erythroid cells, where the expression of both the uniporters Mitoferrin1/2 [125–127] and the “kiss and run” mechanism have been described [128, 129] (Fig. 4). More precisely, the so called “kiss and run” mechanism consists in delivering of iron to mitochondria by the direct interaction of Tf-containing endosomes with the organelle. Recently, by super-resolution three-dimensional direct stochastic optical reconstruction microscopy Das and colleagues defined that Tf-containing endosomes directly interact with mitochondria also in epithelial cells [130] and, more interestingly, that the iron released by Tf regulates the interaction between mitochondria and endosomes [130]. Even if not yet directly confirmed in neuronal cells, these results agree with the previous finding that Tf can be targeted to mitochondria via TfR2 in dopaminergic neurons [131]. This work demonstrated, in animal models and patients, that iron accumulation in dopaminergic neurons is accompanied by increased Tf levels [131]. These data may be interpreted as a continuous request for iron entry into mitochondria, despite the presence of high cellular iron levels, due to the inefficient production of ISCs, which are cofactors in several biological processes. They are essential for the function of Krebs cycle enzymes and for electron transport through respiratory chain complexes. ISCs are needed for several enzymes that process nucleic acids, such as helicases, DNA polymerase and DNA repair enzymes [132, 133]. The production of ISCs directly affects the regulation of iron metabolism, regulating the activity of IRP1 protein [134, 135]. It has also been proposed that ISC proteins may act as sensors of mitochondrial iron status; thus, defects in ISC or heme production might be a general mechanism for the development of iron overload as an effect of the cell needing to revert the lack of these important molecules. Indeed, defects in the synthesis of ISC or heme can have serious consequences on health [136–138]; an example is Friedreich’s ataxia (FRDA), the most frequent form of ataxia. This condition is caused by GAA expansion in *FXN*, which severely lowers iron-chaperone frataxin levels [139, 140]. This protein plays a key role in delivering iron to the ISC complex machinery. A second example is a rare disease known as sideroblastic anemia with X-linked ataxia (XLSA/A), which is caused by defects in *ABCB7*, the mitochondrial transporter of the cytosolic ISC precursor [141], which is essential for the maturation of cytosolic ISC proteins. This condition reflects the importance of the mitochondrion in the synthesis of ISC and in maintaining cellular homeostasis.

Another important point is the susceptibility of ISCs to oxidant species, which can be easily generated in mitochondria as byproducts of respiratory activity. ROS can induce the release of iron from mitochondrial ISC proteins of the respiratory chain, which will lead to further ROS production, establishing a vicious self-maintaining cycle.

Thus, the disruption of iron homeostasis can interfere with mitochondrial functions and, consequently, fuel the progression of neurodegenerative mechanisms. Conversely, the alteration of mitochondrial functions may affect mitochondrial iron homeostasis, leading to neurodegeneration. The latter scenario is the one in PANK-associated neurodegeneration (PKAN), one of the most frequent forms of NBIA (Table 1), in which the alteration of PANK2 impairs coenzyme A (CoA) biosynthesis [13, 142]. PKAN usually manifests in early childhood with gait disturbances and rapidly progresses to a severe movement deficit with dystonia, dysarthria and dysphagia. The hallmark of this disease is the eye-of-the-tiger signal in the globus pallidus on T2-weighted MRI due to severe iron accumulation, which is related to neural damage and mitochondrial lesions [143]. The pathogenetic mechanism of PKAN is still not completely clear; however, studies on fibroblasts, induced neurons and astrocytes derived from PKAN patients have highlighted the main role of mitochondria in triggering pathological events [144–148]. These data revealed that the energetic failure detected in these cellular models is associated with oxidative damage and defects in heme and ISC biosynthesis, relating iron dyshomeostasis and CoA defects. Further progress was obtained from PKAN iPS-derived astrocyte models that showed severe iron accumulation and signs of ferroptosis, recapitulating the human phenotype. Interestingly, they were prone to develop a reactive stellate phenotype, gaining neurotoxic features [147]. The severe iron overload detected in PKAN astrocytes has been hypothesized to be due to CoA-dependent impairment of endocytic vesicular trafficking [149], and it might be responsible for the initiation of a cascade of events that leads to neuronal death. Indeed, defects in TfR recycling were established to be a common anomaly in fibroblasts from different subtypes of NBIA patients [150, 151], suggesting impaired iron incorporation as a shared mechanism responsible for iron overload in these pathologies.

Some mitochondrial abnormalities have also been found in other forms of NBIA. In MPAM (Table 1) models, an alteration of calcium homeostasis within the mitochondria has been identified [152]. This promotes an increase in H_2_O_2,_ which, through the Fenton reaction, can lead to ROS formation [153]. A destruction of the cristae of the inner mitochondrial membrane has also been described in PLAN (Table 1) fibroblast patients [154], which can lead to the total degeneration of the organelle. The latter morphological aspect is one of the main characteristics that define ferroptotic cells, and it might be a common feature in all NBIAs, such as PLAN or MPAN, where lipid metabolism disturbances have been shown.

The analysis of fibroblasts and iPS-derived midbrain neurons from BPAN (Table 1) patients revealed that the loss of function of WDR45, involved in autophagic fluxes, had consequences on the mitochondrial network. The obtained data showed an increase in the number of fragmented mitochondria, a decrease in mitochondrial membrane potential, a reduction in ATP production and elevated levels of superoxide dismutase 2, which implies the presence of a large quantity of ROS [155]. In addition, these models showed decreased levels of lysosomal proteins and enzymes and altered autophagy, suggesting that increased cellular iron levels and oxidative stress are accompanied by mitochondrial abnormalities, autophagic defects, and diminished lysosomal function [155, 156].

## Iron deficiency and neurodegeneration

Iron restriction has been mainly associated with alterations in cognitive functions and psychomotor development [157–159]. In these conditions, many important processes, such as decreased myelin synthesis, impaired synaptogenesis, the alteration of neurotransmitter homeostasis and a decline in basal ganglia function, compromise neurodevelopment [160, 161].

Recently, a case of a patient affected by functional iron deficiency and severe neurological and extra-neurological features was described [15]. This patient carries biallelic mutations in *IREB2*, causative of the absence of IRP2 protein, and shows disabling progressive neurodegeneration and microcytic hypochromic anemia. The clinical and cellular phenotypes of the patient recapitulated the neurological and hematological defects previously described in *Ireb2*^−/−^ mice [162, 163], where the lack of IRP2 results in progressive neurodegeneration. Biochemical studies of the patient’s lymphoblastoid cell lines showed functional iron deficiency, altered posttranscriptional regulation of iron metabolism genes, and mitochondrial dysfunction [15]. The authors argued that the cellular deficient phenotype is established by the decreased cellular iron uptake by TfR1 and the concomitant iron sequestration by ferritin. The cases of two other patients carrying complete *IREB2* loss-of-function mutations and affected by severe progressive neurodegeneration and hematological defects have been reported in the literature [16, 164], confirming the relationships between iron deficiency status and the neurodegenerative process.

Another indication of the involvement of iron deficiency and the alteration of the dopaminergic system is the peculiar case of a patient carrying a loss-of-function mutation in L-ferritin who was affected by idiopathic generalized seizures and atypical restless leg syndrome [165]. The analysis of patient primary fibroblasts and iPS-derived neurons revealed a ferritin molecule expressing only H-chains. The augmented avidity of this type of ferritin for iron increases iron incorporation into the protein, leading to decreased cellular iron availability. Interestingly, in these cellular models, diminished levels of cytosolic catalase and SOD1, enhanced ROS production and higher levels of oxidized proteins emerged, suggesting that iron deficiency can also lead to oxidative damage [165], and even if not sufficient to trigger neurodegeneration, it can promote alterations in normal brain function.

Further indirect evidence of the negative effect of iron restriction comes from the results of a large multicenter, phase 2, double-blind FAIRPARK-II trial of 372 PD patients [166]. The patients were enrolled for early diagnosis and never treated with L-DOPA. Despite the evidence of brain iron removal by chelators, the group of patients treated with DFP suffered from a negative clinical outcome [166]. This was attributed to the effect of iron chelation on dopamine synthesis due to the inhibition of the activity of the iron-dependent tyrosine hydroxylase. However, it also suggests that the removal of brain iron excess, even if obtained with an iron-redeployed-chelator, might equally induce iron restriction for neuronal cells [167].

## Iron in psychiatric disorders

Iron is also associated with brain disease not strictly defined as neurodegenerative ones. In the field of psychiatry, for instance, the use of MRI has revealed that lower-than-normal iron levels in the basal ganglia and thalamus are positively associated with psychotic and schizotypal symptoms in Early Psychotic Spectrum Disorders [168]. Similarly, lower iron concentrations in striatal regions in depressed patients correlate with a decline in cognitive-affective functions [169]. However, it is important to note that these measurement techniques can capture only specific iron configurations (i.e., when bound to ferritin) [170]. Lotan and colleagues [171], on the other hand, by directly quantifying total iron and ferritin on post-mortem specimens, observed that despite lower ferritin levels, total iron is higher in schizophrenic subjects compared to controls in the prefrontal cortex [171]. Additional considerations are therefore necessary when attributing iron dysregulation in mental disorders. In any case, iron plays a role in various psychiatric pathologies. Iron is crucial for neurotransmitter synthesis and particularly interacts within the dopaminergic pathway [172]. Even in different forms of NBIA, the iron accumulation has been observed not only in the area responsible for parkinsonian symptoms but also in areas primarily innervated by the dopaminergic system [13]. The issue is not limited to excess iron; iron deficiency also leads to alterations in dopaminergic receptors [173]. In addition, iron is not only important for the dopaminergic pathway but also plays a “synaptic” role. Chelating iron not only reduces synaptic transmission activity in hippocampal slices but also partly hinders long-term potentiation (LTP), while an increase in iron concentration facilitates LTP [174]. Under physiological conditions, spatial memory training increases DMT1 expression in the rat hippocampus [175], favoring cellular iron incorporation. Iron uptake, when NMDA receptors are stimulated, serves to generate RyR-mediated calcium signals through the production of ROS [174]. This localized increase in calcium in dendritic spines and dendrites [176] may have a significant role in NMDA spikes, which are fundamental processes in cognition, perception, and learning [177]. A dysregulation in the “synaptic” iron pathway could also lead to ferroptosis, as the inhibition of GPX4 causes dendritic damage, lipid peroxidation, and cell death, albeit partially attenuated by inhibiting RyR-mediated Ca^2+^ release [178].

## Conclusions

The role of iron in neurodegeneration has been debated for a long time. Even if indirectly involved, the toxicity that iron exerts at a neuronal level is devastating. The main physiological processes, including the maintenance of redox status, proteolytic control, energy production, and membrane fluidity, are compromised by iron imbalance. Iron overload appears to induce an auto-toxic circuit resulting in neurodegeneration, but iron deficiency has also been implicated in neuronal death. Thus, it is extremely important to clarify the association between the neurodegenerative process and the mechanisms concerning iron dysmetabolism. A greater understanding of the physiological and pathological mechanisms involved could allow the development of new effective therapies for patients affected by neurodegenerative diseases. Currently, there are no effective treatments to reverse the neurodegenerative process, and the cures are mainly symptomatic. Different therapeutic approaches have been studied to avoid iron accumulation and its consequences. DFP was used in several clinical trials on PD [179–181], AD [182, 183], FRDA [184, 185], PKAN [186–188], and NF [106], with only a few cases of positive clinical outcomes [106, 184]. Therefore, evidence that avoiding iron imbalance reverses the pathological mechanism of the disease is still lacking, except for some cases of symptoms stabilization [106, 187]. Given that the iron chelator does not modify the diseases suggests the noncausal role of iron in most neurodegenerative diseases, but it should be kept in mind that the iron accumulation process is very slow, and when it becomes evident, neuronal death has already occurred. Therefore, treatment with chelators is performed when the damage is already severe and difficult to recover. An alternative explanation for the limited success of chelation therapies can be ascribed to the involvement of multiple iron roles: iron assumes a crucial role not only in neurotransmitter synthesis, primarily dopamine, but also in synaptic plasticity. Disrupting concurrently these two pathways, it is not surprising that improvements are not observed, but rather cognitive deterioration occurs. In the case of PD, better results might be obtained through the concurrent administration of L-DOPA with an iron chelator, as it helps to balance the dopaminergic pathway [179], which would otherwise be disrupted by iron deficiency [166, 167]. Another factor to consider is that even low ferritin levels might actually hide a high amount of redox-active iron [171], which triggers ferritin oxidation and its massive precipitation [93]. Therefore, a low MRI ferritin-signal does not exclude the presence of significant neurotoxic iron. Currently, it is challenging to provide definitive therapeutic recommendations because the understanding of how iron interacts individually with mitochondria, dopamine, and synapses, as well as how these three systems interplay in situations of iron dyshomeostasis, remains incomplete. These disappointing results are stimulating new therapeutic approaches aimed at limiting iron overload and its consequences. Compounds with multiple functions that can block several steps of the neurodegenerative process are being tested in preclinical model [189, 190]. In addition, advances in the knowledge of ferroptosis have led to the identification of numerous inhibitors of this process that can be considered novel potential pharmacological targets for neuroprotective strategies [191]. Nonetheless, further studies are needed to elucidate the aspects of this still unclear but extremely complex and interesting relationship between iron and neurodegeneration.
